# Regions outside the DNA-binding domain are critical for proper *in vivo* specificity of an archetypal zinc finger transcription factor

**DOI:** 10.1093/nar/gkt895

**Published:** 2013-10-06

**Authors:** Jon Burdach, Alister P. W. Funnell, Ka Sin Mak, Crisbel M. Artuz, Beeke Wienert, Wooi F. Lim, Lit Yeen Tan, Richard C. M. Pearson, Merlin Crossley

**Affiliations:** ^1^School of Biotechnology and Biomolecular Sciences, University of New South Wales, NSW 2052, Australia and ^2^School of Molecular Bioscience, University of Sydney, NSW 2006, Australia

## Abstract

Transcription factors (TFs) are often regarded as being composed of a DNA-binding domain (DBD) and a functional domain. The two domains are considered separable and autonomous, with the DBD directing the factor to its target genes and the functional domain imparting transcriptional regulation. We examined an archetypal zinc finger (ZF) TF, Krüppel-like factor 3 with an N-terminal domain that binds the corepressor CtBP and a DBD composed of three ZFs at its C-terminus. We established a system to compare the genomic occupancy profile of wild-type Krüppel-like factor 3 with two mutants affecting the N-terminal functional domain: a mutant unable to contact the cofactor CtBP and a mutant lacking the entire N-terminal domain, but retaining the ZFs intact. Chromatin immunoprecipitation followed by sequencing was used to assess binding across the genome in murine embryonic fibroblasts. Unexpectedly, we observe that mutations in the N-terminal domain generally reduced binding, but there were also instances where binding was retained or even increased. These results provide a clear demonstration that the correct localization of TFs to their target genes is not solely dependent on their DNA-contact domains. This informs our understanding of how TFs operate and is of relevance to the design of artificial ZF proteins.

## INTRODUCTION

Transcription factors (TFs) are typically regarded as having two distinct components: a sequence-specific DNA-binding domain (DBD) and a *trans*-acting functional domain that is capable of activating or repressing gene expression. Under this model, the DBD acts to direct the TF to certain regulatory regions in the genome based on its affinity for a particular DNA sequence and the *trans*-acting domain then imposes regulatory effects on the appropriate gene. Recognizing the capability of the two distinct domains to function autonomously has been helpful in understanding TF function and has led to the development of methodologies such as the yeast two-hybrid system, where two separable domains are reunited to recreate a functional TF. Nevertheless, it is known that the situation is sometimes more complex: DNA-binding domains can also make functional protein–protein interactions with coregulators, and several results imply that non-DNA-binding domains can contribute to the localization of TFs to their target genes (1–3).

Most strikingly, it is now becoming clear that the DBDs of TFs alone are unlikely to provide sufficient specificity to account for the highly limited *in vivo* genomic profiles being observed in chromatin immunoprecipitation followed by high-throughput sequencing (ChIP-seq) experiments. In these experiments, the genome-wide occupancy of TFs is determined by immunoprecipitating them together with associated DNA fragments and then identifying those fragments via large-scale sequencing. The TFs are first cross-linked to their target sites in living cells so that *in vivo* maps of binding sites can be generated. These maps show that *in vivo* TFs are far more discriminating about where they bind than *in vitro*. That is, *in vitro* most TFs bind to all sites that reasonably match their consensus binding sequence but *in vivo* only a small subset, sometimes <1% of possible sites are bound. For instance, ChIP-seq studies have revealed that GATA-1 binds to <1% of predicted consensus sites in erythroid cells (4). The poor correlation between predicted and observed occupancy has been dubbed the ‘futility theorem’ by one group of authors based on the assertion that essentially all *in vivo* TF binding site predictions generated using binding consensus sequences for individual TFs will have no functional role (5).

At the root of this problem is the length of the DNA-binding motif and the information content contained therein. Given the size of the human genome (∼3.9 gigabases), a motif would need to be >16 bp in length to be unique if a random nucleotide distribution is assumed. Despite this, most eukaryotic TF motifs are rather short and only some positions carry strong sequence preference. The zinc finger (ZF) TFs of the Krüppel-like factor (KLF) family, for instance, recognize a 10-bp sequence with only four of these positions being restricted to a single specific nucleotide (6,7). Furthermore, the overall motif is mostly composed of C and G nucleotides, which are over-represented in promoter regions. Taken together, these observations point to a level of specificity far short of what might be expected. It does not seem that the DNA-binding surface within the ZF domain alone could provide sufficient specificity to explain observed binding *in vivo*. Thus, other regions, such as those outside the DBD, or other phenomena, such as the availability of target sites, may also play a role.

We have focused on ZF domains, the most prevalent DBD in the proteome, to gain a better understanding of the mechanisms by which ZF TFs are localized to particular *cis*-regulatory elements. KLF3 is an archetypal ZF protein belonging to the SP/KLF family and nine SP and 17 KLFs have been described to date. These proteins are characterized by a highly conserved C-terminal DBD composed of three tandem classical ZF motifs and variable N-terminal domains that can recruit coactivators or corepressors (8). Several artificial TFs have been modeled on this family, comprising N-terminal functional domains fused to three C-terminal ZFs (9).

KLF3 has known roles in adipogenesis, erythropoiesis and lymphopoiesis [reviewed in (10)]. The molecular mechanisms by which KLF3 regulates gene expression have been extensively investigated. KLF3 uses its N-terminal non-ZF domain to recruit the corepressor C-terminal binding protein (CtBP) (11). CtBP in turn can recruit a range of factors including histone methyltransferases, histone deacetylases and histone-lysine-specific demethylases (12–15) that remodel chromatin to repress gene expression. Thus, KLF3 can be regarded as a typical ZF TF with an N-terminal functional domain and a C-terminal DBD.

In this work, we have analyzed the *in vivo* DNA-binding specificity of KLF3 using ChIP-seq. We have also tested a point mutant that is intact except for a two amino acid change in its N-terminal domain that abrogates binding to the cofactor CtBP. In addition, we have assessed the contribution of the entire N-terminal non-ZF domain by examining a deletion mutant that lacks this domain, and thus consists only of the ZF domain and an adjacent putative nuclear localization sequence.

The results obtained define for the first time the *in vivo* binding consensus of KLF3 and show that it conforms to the site previously identified for other family members, KLF1 and KLF4. We have also further refined the KLF binding consensus and identified additional nucleotide positions within it that influence DNA-binding specificity. We show that KLF3 preferentially binds at proximal promoter elements. Most importantly, the work with the mutants demonstrates that the N-terminal domain contributes to *in vivo* binding site selection, as the ZF domain alone is unable to localize to a large proportion of the binding sites and also appears to bind to new sites. The mutant unable to bind CtBP shows an intermediate pattern, suggesting that contact with CtBP also influences occupancy but is not the sole additional determinant in specifying DNA binding. Taken together, the results demonstrate that ZF domains alone are insufficient for specifying the *in vivo* genomic binding profiles of ZF TFs.

## MATERIALS AND METHODS

### Generation of cell lines

*Klf3*^−^*^/^*^−^ murine embryonic fibroblast (MEF) cell lines were generated from *Klf3*^−^*^/^*^−^ mice as previously described (16). All cells were cultured in Dulbecco’s modified Eagle’s medium (DMEM) supplemented with 10% FCS and 1× penicillin, streptomycin and glutamine (Cat# 10378-016 Life Technologies, Carlsbad, CA, USA). *Klf3*^−^*^/^*^−^ MEFs were then transduced using the Murine Stem Cell Virus Retroviral Expression System (Clontech Laboratories, Mountain View, CA, USA) with either *Klf3-V5*, *ΔDL-V5* or *DBD-V5*. Stable clones expressing each transgene were then isolated under puromycin selection (2 μg/ml) using the cell dilution method in 24-well plates. Single clones were evaluated for relative protein expression by western blot using anti-V5 antibody (Cat# R960-CUS, Life Technologies Carlsbad, CA, USA) and for relative mRNA expression by real-time reverse transcriptase-polymerase chain reaction (RT-PCR) (17). For western blots molecular weight markers and protein bands were imaged by light and chemiluminescence, respectively. Oligonucleotide sequences for real-time RT-PCR are available in Supplementary Table S1. Electrophoretic mobility shift assay (EMSA) was performed as previously described (18). Probe sequences for EMSA are available in Supplementary Table S2.

### Chromatin immunoprecipitation

ChIP was conducted in duplicate on *Klf3*^−^^/−^ MEFs expressing recombinant *Klf3-V5*, *ΔDL-V5* or *DBD-V5*. Approximately 5 × 10^7^ cells were used for each experiment and ChIP was conducted as previously described (19) using an anti-V5 antibody (Cat# R960-CUS, Life Technologies, Carlsbad, CA, USA). Quantitative real-time PCR was performed on ChIP material using the primers in Supplementary Table S3. Library preparation was performed using the TruSeq DNA Sample Preparation Kit (Cat# FC-121-2001, Illumina, San Diego, CA, USA) according to the manufacturer’s instructions with minor modifications. Adapter sequences were diluted 1/40 before use and following adapter ligation, the library size extracted from the gel was 100–280 bp (excluding adapters) in line with the size of sonicated fragments. Library preparation was performed by the Ramaciotti Centre, University of New South Wales, New South Wales, Australia

### Sequencing

Libraries (6 inputs and 6 IP samples) were multiplexed into four lanes using sample-specific adapters such that there were three samples per lane. Samples were sequenced using 50 bp chemistry on the HiSeq 2000 (Illumina, San Diego, CA, USA). Library preparation and sequencing were performed by the Ramaciotti Centre, University of New South Wales, New South Wales, Australia. Quality control on the sequence data was performed using FastQC v0.10.1 available from http://www.bioinformatics.babraham.ac.uk/projects/fastqc/.

### Alignment

Reads were aligned to the mm9/NCBI37 *Mus musculus* genome using Bowtie2 v2.0.0-beta7 (20). In the first round, Bowtie2 was set to –very-sensitive and –D 40. Non-aligned reads were subjected to a second round of alignment where the read could be soft-clipped by running Bowtie2 with the switch –very-sensitive-local. Resulting alignments were sorted, merged and indexed using Samtools v0.1.18 (21).

### Peak calling, peak overlap and genomic annotation

Peak calling and downstream analysis was primarily performed using the HOMER software package v4.1 (available from http://biowhat.ucsd.edu/homer/ngs/index.html) (22). The script findPeaks.pl was used to for peak discovery using the paired input sample as a control with the settings -style factor, -F 5 and -L 5, requiring 5× fold enrichment over input and 5× fold enrichment over background (surrounding 10 kb) to call a peak. Peaks were subjected to a false discovery rate cutoff of 0.001. Peaks were merged using mergePeaks using the switch -d meaning that peaks had to literally overlap in genomic space to be considered overlapping. Peak lists were annotated using annotatePeaks.pl using the HOMER annotation set for mm9/NCBI37. HOMER was also used to determine sequence conservation around peaks using the mouse PhastCons data supplied with the software package.

### Quantification of ChIP tags

HOMER was used to quantify ChIP tag density at peak locations across the genome. Unless otherwise noted, tags were counted within 400 bp around the peak center (as peak widths could vary across the three different samples). All tag counts were normalized to 100 M reads, and were thus expressed as reads/100 M reads to allow comparison across samples. Histograms of tag densities around various genomic features were also derived using HOMER. Bin sizes varied depending on the application and are given with each result.

### Visualization

HOMER was used to create bedgraph files using the makeUCSCfile program. These were viewed using IGV v2.2 (23). Venn diagrams were produced using BioVenn (24), Venn Diagram Plotter v1.4.3740 (available from http://omics.pnl.gov/software/VennDiagramPlotter.php) and eulerAPE v2.0 (available from http://www.eulerdiagrams.org/eulerAPE/).

### ENCODE data sets

An ENCODE DNase-seq data set produced from murine lung fibroblasts by the Stamatoyannopoulos Laboratory at the University of Washington was downloaded from GEO (Accession# GSM1014199) (25,26). An ENCODE RNA-pol II ChIP-seq data set produced from MEFs by the Ren Laboratory at the Ludwig Institute for Cancer Research was also downloaded from GEO (Accession# GSM918761) (25,27). The raw sequencing reads from these data sets were processed using the ChIP-seq pipeline described earlier in text to make bedgraph files for visualization in IGV and to quantify sequencing tags at genomic locations of interest.

### *De novo* motif analysis

Sequence databases were created from the 100 bp surrounding the centers of peaks using the HOMER script findMotifs.pl. *De novo* motif discovery was conducted on these sequence databases using MEME v4.9.0 (28). The KLF3 motif defined by MEME was fed back into findMotifs.pl to search for instances of known motifs within KLF3 peaks. The position weight matrices of other KLF motifs were extracted from the HOMER motif database. These motifs were visualized using Weblogo v3.3 (29).

### Known motif analysis

The enrichment of known motifs in KLF3 peaks was determined using the findMotifsGenome.pl script in the HOMER package (22).

### Microarrays

Total RNA was purified from *Klf3*^−^^/−^, or *Klf3-V5* rescued MEF cells using tri-reagent according to the manufacturer’s instructions (Sigma-Aldrich, St Louis, MO, USA). RNA was subsequently ethanol precipitated and washed with 75% ethanol in DEPC-treated deionized water for further purification. RNA was then subjected to whole transcript sense labeling and hybridized to Affymetrix GeneChip 1.0 ST mouse gene arrays (Affymetrix, Santa Clara, CA, USA). Microarray preparation and scanning were performed by the Ramaciotti Centre, University of New South Wales, New South Wales, Australia. Microarray data were analyzed using Partek genomic suite v6.5 (Partek Inc., St. Louis, MO, USA).

Microarray CEL files were imported into Partek and normalized using the robust multi-array average algorithm. After confirming array quality (Affymetrix built-in controls and principal components analysis), differential gene expression was calculated and tested for significance using a one-way analysis of variance. Gene expression *P*-values were corrected for multiple testing using a false discovery rate threshold of 0.2.

## RESULTS

### Establishment of a system to compare the binding of normal and two mutant forms of KLF3

**Figure 1.**
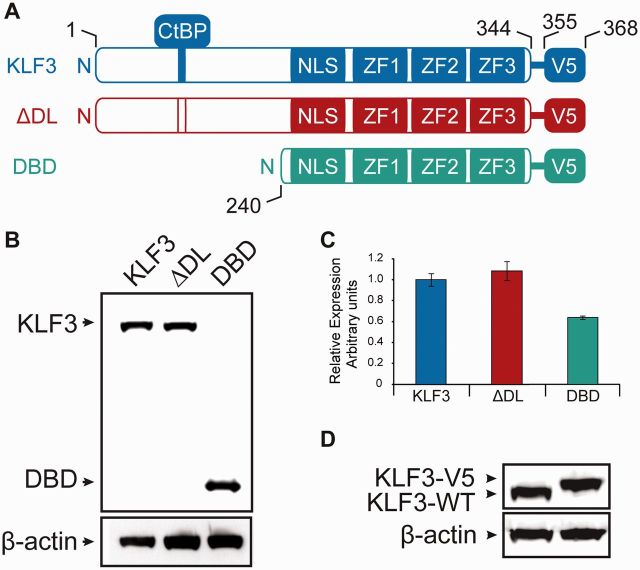
Experimental model for investigating KLF3 occupancy. (**A**) Schematic showing the three constructs used to rescue *Klf3*^−/−^ MEFs. (**B**) Western blot and (**C**) real-time RT-PCR showing relative levels of ectopic protein and mRNA expression of the three constructs in rescued *Klf3*^−/−^ MEFs. For real-time RT-PCR, expression has been normalized to 18 S rRNA and is shown relative to the KLF3 rescue, which has been set to 1.0. Shown are the means of either two (ΔDL and DBD) or three (KLF3) independent experiments. Error bars represent standard deviation. (**D**) Western blot showing endogenous KLF3 in *Klf3*^+/+^ MEFs and ectopic KLF3 in *Klf3*^−/−^ MEFs recued with *Klf3-V5*.

We developed a system to compare the occupancy of wild-type KLF3 with two KLF3 mutants (Figure 1A). The first mutant, designated ΔDL, contained a two amino acid substitution with AS replacing DL in the CtBP-contact motif—PVDLT—within the N-terminal domain of KLF3. This mutation effectively renders KLF3 unable to recruit the corepressor CtBP (11). The second mutant, designated DBD, involved the deletion of the entire N-terminal domain, leaving just the putative nuclear localization signal and the ZF DBD intact. All three constructs were tagged with a V5 epitope via a glycine–serine linker to enable consistent immunoprecipitation and comparison between samples.

To avoid any competition between the mutants and endogenous, wild-type KLF3 we used MEFs generated from *Klf3*^−^*^/^*^−^ mice (30). These *Klf3*^−^*^/^*^−^ MEFs were rescued with each of the three *Klf3* constructs using the MSCV retroviral delivery system and stable clonal cell lines were generated expressing wild-type or mutant *Klf3*. Cell lines were examined for expression of *Klf3*, *ΔDL* and *DBD* mRNA and protein. Cells expressing similar levels of protein were selected for further analysis (Figure 1B). In these lines, we found equivalent expression of *Klf3* and *ΔDL* mRNA, whereas levels of *DBD* mRNA were somewhat lower (Figure 1C). Importantly, the level of ectopic KLF3 protein was also shown to be similar to the level of endogenous KLF3 in wild-type MEFs (Figure 1D). Immunofluorescence confocal microscopy confirmed that the KLF3 wild-type and mutant proteins were all correctly localized to the nucleus of MEF cells (Supplementary Figure S1).

We also investigated *in vitro* binding of the three constructs to a previously validated CACCC-box probe derived from the *Klf8* promoter (31). In EMSA experiments, we saw equivalent binding for KLF3 and ΔDL, whereas DBD interacted more strongly with the *Klf8* probe (Supplementary Figure S2). ChIP-seq was then performed on MEF cells expressing *Klf3*, *ΔDL* or *DBD* in duplicate. Samples were sequenced on the HiSeq 2000 (Illumina, San Diego, CA) using 50 bp chemistry. Across the six samples, a total of >700 M reads were mapped to the mouse genome using Bowtie2 (for details see Supplementary Table S4). Peaks for each mutant were called on individual replicates, and the overlap between replicates was established (Supplementary Figure S3) with overlapping peaks taken forward for further analysis. An annotated table containing ChIP peaks across all three samples and sorted by KLF3 peak height can be found in Supplementary Table S5. Replicates were shown to be consistent based on the correlation between peak heights across replicates at overlapping peak locations (Supplementary Figure S4).

Encouragingly, the ChIP-seq peak analysis revealed strong peaks at previously identified KLF3 targets including *Klf8*, *Lgals3* and *Fam132a* (31–34). To further validate these results, a number of peaks were selected for confirmation by ChIP–PCR. These peaks included the known targets mentioned earlier in the text, a new peak at the *Stard4* promoter and two previously established unbound regions in the *Klf8* locus (31). New independent ChIP assays were performed on each of the three cell lines in duplicate and the recovered DNA was subjected to amplification by quantative real-time PCR using primers for the six specific loci (primer sequences available in Supplementary Table S3). As shown in Supplementary Figure S5, the ChIP–PCR confirmed the presence of the expected peaks and the absence of peaks in negative control regions. To further validate the biological relevance of the rescued cell lines, we examined endogenous KLF3 occupancy at a number of genomic loci where we observed KLF3-V5 peaks by ChIP-Seq. These ChIP experiments confirmed that the sites identified as targets of KLF3-V5 are also bound by endogenous KLF3 (Supplementary Figure S6).

### The genomic binding profile of full length KLF3

First the binding profile of wild-type full length KLF3 was analyzed as a reference for comparison with the mutants. A total of 14 115 KLF3 peaks were identified as overlapping across the two replicate sets of samples (Supplementary Figure S3). The distribution of KLF3 peaks in promoters, exons, introns and other regions across the *Mus musculus* (mm9/NCBI37) genome was analyzed based on RefSeq annotations. Promoter regions were defined as being −1 to +0.1 kb from the RefSeq transcription start site (TSS); intronic regions were those lying between exons; and intergenic regions made up the rest of the genome. Peaks that fell into coding exons, 5′ and 3′ UTR exons and close to the transcription termination sites (−100 bp to +1 kb) were all labeled as ‘other’.

**Figure 2.**
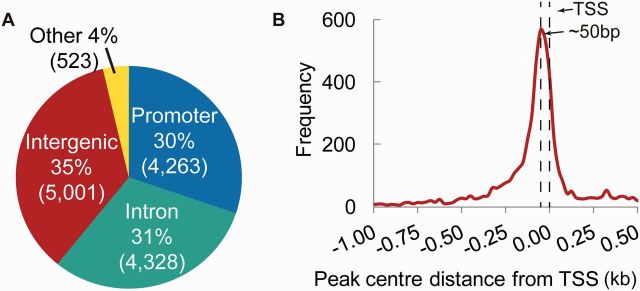
Genomic localization of KLF3 peaks. (**A**) Distribution of KLF3 peaks within different genomic regions. Promoters are defined as the region −1000 bp, +100 bp around the TSS of Refseq genes. Peaks that fell into CDS exons, 5′ and 3′ UTR exons and transcription termination sites (−100 bp to +1 kb) were all labeled as ‘other’. Percentages lying in each region are given, and absolute peak numbers are shown in parentheses. (**B**) Histogram of peak centers within 1.5 kb of the Refseq TSS with 20 bp bins.

Just under one-third of the peaks were found to lie in promoters, approximately one-third in introns and just over one-third in intergenic regions (Figure 2A). As promoters and introns constitute much less than a third of the total genome each, these results represent a strong enrichment of KLF3 peaks in promoters and also a notable but lesser enrichment in introns. The precise location of the promoter peaks relative to the TSS is shown in Figure 2B. The confluence of peak centers is located ∼50 bp upstream from the TSS. The remarkable proximity of KLF3 peaks to the TSS fits with results from *Drosophila*, where it has been suggested that the cofactor CtBP functions as a short-range corepressor for the TFs Krüppel, Knirps, Giant and Snail and is typically found within 100 bp of promoters or of activating TFs within enhancer units (35–37).

The conservation of nucleotides around KLF3 peaks was also analyzed using the 30-way vertebrate PhastCons data from UCSC (Supplementary Figure S7A). An enrichment of conserved nucleotides around the center of KLF3 peaks is evident, indicating that these sites are more evolutionarily constrained than the surrounding sequence, and therefore likely to be functional regions. Higher conservation is observed for promoter peaks than intronic and intergenic peaks.

### Colocalization of KLF3 peaks and other promoter marks

We sought to supplement our KLF3 data by incorporating two data sets from the ENCODE consortium. An RNA-pol II ChIP-seq data set from MEFs produced by the Ren Laboratory at the Ludwig Institute for Cancer Research was analyzed using our ChIP-seq pipeline to produce bedgraph files for visualization (25,27). Similarly, a DNase-seq data set produced by the Stamatoyannopoulos Laboratory from mouse lung fibroblasts at the University of Washington was also analyzed (25,26). These DNase-seq data were used to establish the overlap between nucleosome-depleted regions and KLF3 binding sites genome-wide. KLF3 peaks showed a strong enrichment for nucleosome depletion, and splitting KLF3 peaks into subsets based on genomic localization revealed a divergence in the extent of this depletion (Supplementary Figure S7B). KLF3 promoter peaks were found to have almost double the nucleosome depletion compared with peaks in introns or intergenic regions.

### The role of KLF3 in gene regulation

Given that a large number of KLF3 peaks at proximal promoters had been identified, we wished to better understand how KLF3 occupancy related to changes in gene expression. To accomplish this, we performed Mouse Gene ST 1.0 gene expression microarrays (Affymetrix, CA, USA) on *Klf3*^−^^/−^ MEFs and the same cell line rescued with *Klf3-V5*. A *P*-value cutoff of <0.05 was applied using 1-way analysis of variance and transcripts dysregulated more than 2-fold were selected. In total, 196 transcripts were repressed and 201 were upregulated on rescue with KLF3 according to these cutoffs. A volcano plot of these data is found in Supplementary Figure S8, and the microarray data are available in Supplementary Table S6.

**Figure 3.**
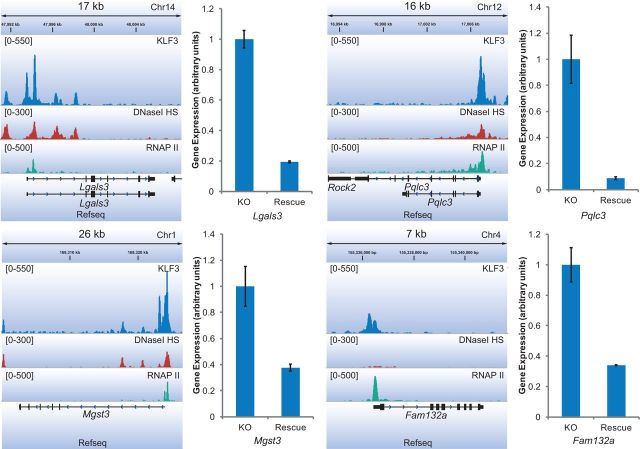
A selection of putative KLF3 target genes. Genes displayed were repressed >2-fold on rescue with KLF3 and also exhibit a ChIP peak at the proximal promoter. DNase-seq and RNAP II ChIP-seq tracks generated from experiments from the Ren and Stamatoyannopoulos Laboratories, respectively, are also displayed. Both were sourced from the ENCODE project (25–27). Gene expression changes are based on microarray data and have passed a *P* < 0.05 cutoff as measured by one-way analysis of variance. Error bars represent standard error of the mean.

To further refine these putative KLF3 targets, we searched within these groups for genes that exhibited a KLF3 peak at the proximal promoter (−1 kb, +0.1 kb) (Supplementary Table S5). A total of 65 genes showed >2-fold repression in the presence of KLF3 and a KLF3 promoter peak. Only 19 genes showed activation >2.0-fold in the presence of KLF3 and a KLF3 promoter peak consistent with previous results and reinforcing the view that KLF3 is predominantly a repressor of transcription (32). Representative examples of genes repressed in the presence of KLF3 targets are given in Figure 3.

### The KLF3 consensus site conforms to the typical KLF family site

**Figure 4.**
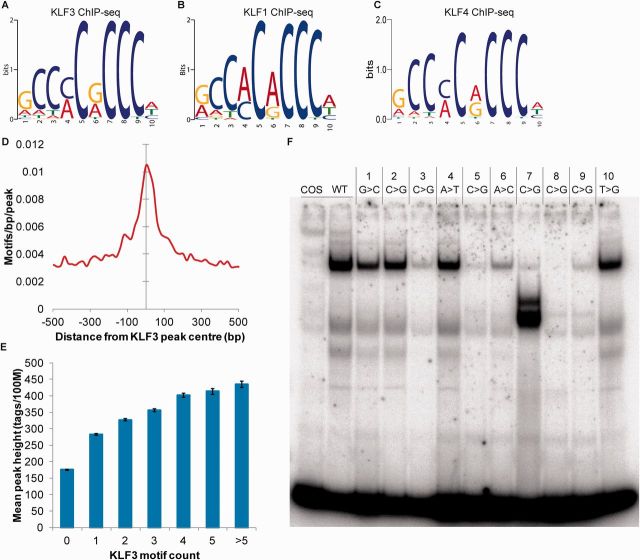
Characterization of the KLF3 consensus DNA-binding site. (**A**) The KLF3 consensus binding site derived from KLF3 ChIP-seq peaks. *De novo* motif discovery was accomplished using MEME on a sequence database composed of the 100 bp surrounding the top 500 peaks ranked by peak height. (**B**) KLF1 consensus binding motif from a ChIP-seq experiment on erythroid cells (38). (**C**) KLF4 consensus binding site from ChIP-seq on ES cells (7). (**D**) Cumulative distribution of KLF3 consensus motifs within KLF3 ChIP-seq peaks. (**E**) Relationship between peak height and KLF3 motif count within the peak. Motif counts were established using HOMER and the mean peak height was taken. Error bars represent the standard error of the mean. (**F**) EMSA showing the effect of mutation of the KLF3 consensus on DNA-binding strength. Point mutations were introduced at each position of a *β-globin* CACCC probe (18) as indicated. COS and WT lanes contain wild-type *β-globin* probe; COS lane contains nuclear extracts from cells transfected with empty pMT3 vector. Probe sequences are given in Supplementary Table S2.

*De novo* motif discovery on KLF3 peaks was accomplished using MEME on the 100 bp surrounding the top 500 peak centers ranked by peak height (28). The motif discovered was highly similar to those previously reported for other KLF TFs based on ChIP-seq experiments (6,7) (Figure 4A–C). Although different KLFs exhibit significantly different biological functions, it appears that these differences are not due to major differences in their preferred DNA consensus sequence. It seems possible that several, if not all KLFs in a cell, might target the same regulatory regions. This notion is supported by the previous observation that in erythroid cells KLF3 can repress a subset of KLF1 target genes (32). We were also interested to see if the KLF3 motif found in promoters differed from the motifs found in intronic and intergenic peaks. Our analysis showed that there was little divergence in motif preference in these different genomic regions (Supplementary Figure S9).

The *de novo* generated KLF3 motif was also used to test the location of the consensus motif within KLF3 peaks and the number of motif occurrences within the peaks. As expected, the motif was found to be centrally enriched within the pooled KLF3 ChIP peaks (Figure 4D). Interestingly, the mean height of KLF3 peaks was found to increase as the number of motifs within a peak increased (Figure 4E), although the relationship was not linear (that is, the presence of two motifs led to much less than a doubling in peak height).

We also investigated the enrichment of known TF motifs within 200 bp of KLF3 peak centers in promoter, intronic and intergenic regions (Supplementary Figure S10). Similar motifs were found to be enriched in both intronic and intergenic KLF3 peaks, where we saw an overrepresentation of binding sites for AP-1, and TEF and RUNX family members. However, KLF3 promoter peaks showed enrichment for a different set of motifs, with the presence of consensus sites for ETS and E2F factors, along with CCAAT binding proteins. Across the three regions, the highest enrichment was for the AP-1 motif at intergenic peaks where 33.29% of KLF3 peaks contained this motif, compared with a background expectancy of 6.57%. The diversity of motifs enriched within KLF3 peaks would suggest that KLF3 may target genomic sequences in a variety of *cis*-regulatory modules to influence gene expression.

### Validation of the *de novo* generated KLF3 consensus binding site

The *de novo* generated motif was highly similar to sequences previously reported for KLF family members. EMSA was used to further evaluate this consensus sequence (Figure 4F), using a previously characterized probe from the *β-globin* locus that conforms to the KLF3 DNA-binding motif (18). The importance of each position was analyzed using a series of probes that introduced point mutations to replace the consensus base with the least preferred alternative. Each of these mutations reduced binding (Figure 4F and Supplementary Figure S11A); in particular, we were able to confirm the importance of the invariant C residues at positions 5, 7 and 8, where the presence of a G on the coding strand presumably allows hydrogen bonding with contact arginine residues in the DBD of KLF3. There is also a strong preference for a C nucleotide at positions 3 and 9. Interestingly, the C to G mutation at position 7 resulted in novel, high affinity binding by an unidentified protein.

In agreement with the consensus, we found greater tolerance at positions 2 and 10, whereas introduction of a C at position 6 had a significant impact on binding. Position 4 appears able to accept a T residue that is not suggested by the consensus. A preference for a G at position 1 was observed in the KLF3 ChIP-seq results; however, the importance of this nucleotide has not been previously discussed or analyzed to our knowledge. When the G at position 1 was changed to a C, a reduction in binding was observed, indicating that the G at position 1 is important (Figure 4F). To investigate this further, we examined KLF3 binding to a series of *β-goblin* CACCC probes containing each of the four possible bases at position 1. In agreement with the consensus, we found a strong preference for G or A at this position (Supplementary Figure S11B). Taken together, these results demonstrate that the *in vitro* binding preference provides support for the ChIP-seq generated consensus and highlights for the first time the importance of having a G or A at position 1.

### Mutations in the non-DBD and their effects on DNA binding

**Figure 5.**
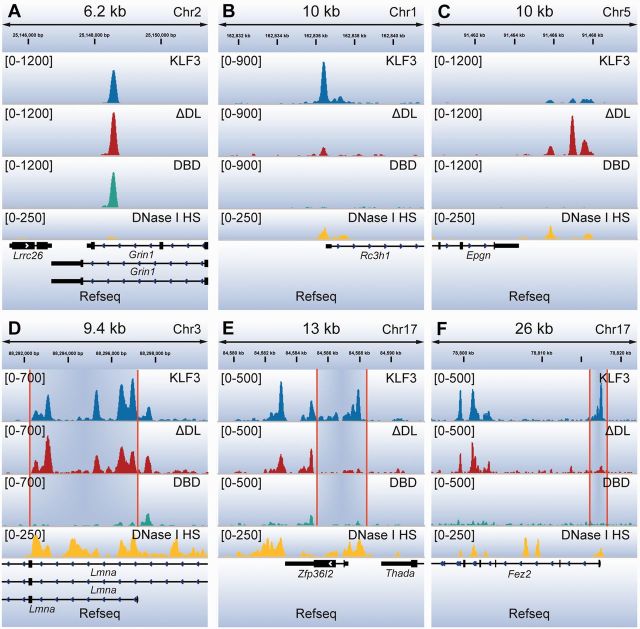
An illustrative range of peaks showing similarities and differences between the occupancy of KLF3, ΔDL and DBD. Notable changes in occupancy are highlighted by red vertical bars.The *y*-axis scales may differ between panels but that within a panel, KLF3, ΔDL and DBD tracks use the same scale to allow direct comparison of peak height. The DNase-seq track was generated from fibroblast data from the Stamatoyannopoulos Laboratory and was sourced from the ENCODE project (25,26).

Having profiled the occupancy of KLF3, we next sought to compare this binding profile against the two mutants: ΔDL and DBD. In total, 12 248 and 4955 peaks were identified for ΔDL and DBD, respectively, compared with the 14 115 peaks identified for KLF3 (Supplementary Figure S3). A range of striking differences was observed in the binding profiles of these proteins and a number of illustrative peaks are displayed in (Figure 5). Panel A shows an example where all three constructs have similar binding profiles in the last intron of *Grin1*. Panel B shows a dramatic loss of binding by ΔDL and DBD at the promoter of *Rc3h1.* Panel C shows a new binding activity by ΔDL that is not present in KLF3 3′ to the *Epgn* gene. Panel D shows loss of binding by DBD in the region marked by red bars, whereas both KLF3 and ΔDL show near identical binding profiles. In panels E and F, ΔDL shows loss of binding solely at the proximal promoter, but maintains downstream peaks in the case of *Zfp36l2* (panel E) and in the body of the *Fez2* gene (panel F), while DBD shows a broad loss of binding across all regions. In summary, our comparative analysis of genome-wide binding by KLF3, ΔDL and DBD revealed that loss of binding by ΔDL at promoters is common and dramatic loss of binding by DBD is almost universal. However, as the examples in Figure 5 show, specific profiles are often complex, with increased binding by ΔDL at some regions and retention of ΔDL and DBD binding at others.

**Figure 6.**
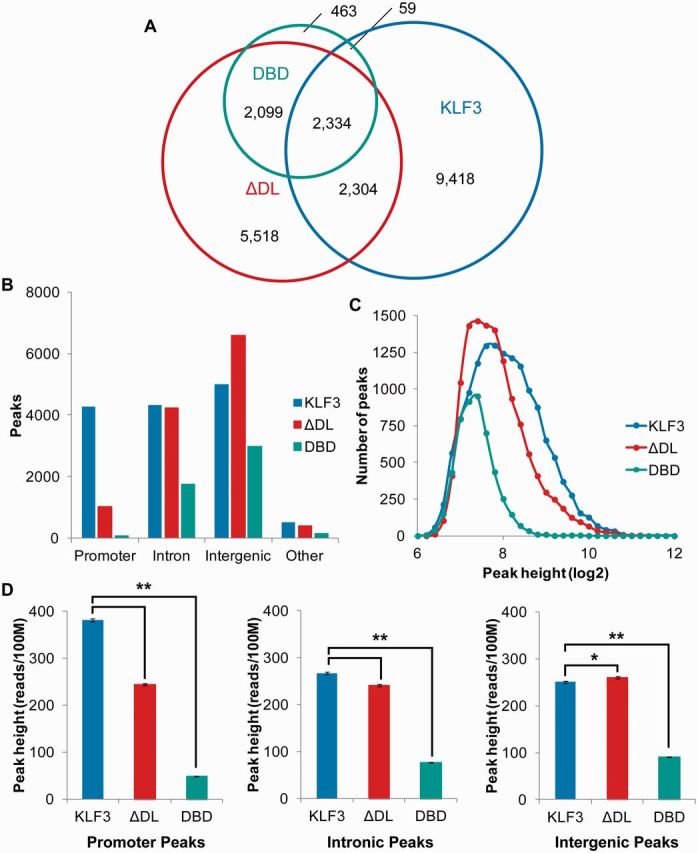
Contrasting KLF3, ΔDL and DBD peaks. (**A**) Proportional Venn diagram showing the overlap of peaks between the three proteins. (**B**) Distribution of KLF3 and mutant peaks across the genome. Promoters are defined as the region −1000 bp, +100 bp around the TSS of Refseq genes. Peaks that fell into CDS exons, 5′ and 3′ UTR exons and transcription termination sites (−100 bp to +1 kb) were all labeled as ‘other’. (**C**) Histogram showing the distribution of peak heights across KLF3 and the two mutants. (**D**) Differences in peak heights between KLF3 and KLF3 mutants at various genomic regions. Values are mean peak heights (reads/100 M reads within 400 bp of peak centers). Error bars represent standard error of the mean. *P < 0.05 **P < 0.00005.

We were interested to see whether KLF3, ΔDL and DBD bound the same sites within the genome. The overlap of peaks between the three mutants is shown in Figure 6A. For peaks to be considered overlapping, their boundaries had to literally overlap in genomic space. KLF3 and ΔDL show some degree of overlap; however, the majority of sites are distinct and non-overlapping. Around half of the DBD peaks overlap with KLF3. ΔDL and DBD show a closer relationship to each other, with the vast majority of DBD peaks co-occurring with ΔDL.

We then investigated whether KLF3, ΔDL and DBD showed equivalent patterns of binding to promoters, introns or intergenic regions. The distribution of KLF3, ΔDL and DBD peaks was analyzed based on genomic region. The raw numbers of peaks occurring within each genomic region are displayed in Figure 6B. It is immediately apparent that there is a dramatic reduction in the number of peaks in the ΔDL and DBD experiments compared with KLF3 at the proximal promoters of genes, consistent with the data shown previously in Figure 5B, E and F. ΔDL shows ∼75% fewer promoter peaks, whereas DBD shows almost no promoter peaks at all (Figure 6B). In intronic and intergenic regions, ΔDL and KLF3 showed similar numbers of peaks, whereas DBD again showed fewer peaks overall.

There was also a striking effect on peak height. It was possible to quantify the number of sequencing tags falling under each peak to compare peak heights in KLF3, ΔDL and DBD samples. Sequencing tags had already been normalized and were expressed as tags per 100 M reads to allow direct comparison between the three experiments. Reads were counted within a 400 bp region surrounding each peak center genome-wide for each of the peak lists generated for KLF3, ΔDL and DBD. A histogram of peak height across the three proteins is presented in Figure 6C. KLF3 exhibits the greatest peak heights—evidenced by the shift of frequency profile to the right. ΔDL shows fewer peaks at higher peak heights but has more peaks showing weak binding than KLF3. DBD exhibits a strong loss of peak height with a large shift to the left. It is also clear that there are far fewer DBD peaks called.

It was then possible to look more closely at the differences in peak heights between KLF3, ΔDL and DBD. The mean peak height of KLF3 was compared with ΔDL and DBD at various genomic regions (Figure 6D). ΔDL shows a smaller mean peak height than KLF3 at the proximal promoter of KLF3-bound genes. DBD shows an even smaller mean peak height at promoters compared with KLF3, reinforcing observations from previous analyses (Figures 5B, E, F and 6B). At intronic and intergenic regions, KLF3 and ΔDL showed similar mean peak heights, whereas DBD exhibited much weaker occupancy. Despite the observed changes in binding *in vivo*, KLF3 and ΔDL both bind to DNA with similar affinity *in vitro* (Supplementary Figure S2). Furthermore, in contrast to its *in vivo* activity, DBD shows a stronger interaction with DNA than full length KLF3, such that progressive deletion of the N-terminal domain of KLF3 appears to result in increased DNA-binding *in vitro* (Supplementary Figures S2 and S12).

We also examined the KLF3, ΔDL and DBD peaks to see whether there were any differences in the KLF3 or KLF3 mutant consensus binding motifs (Supplementary Figure S13). Motifs were similar between KLF3 and ΔDL, although only 142/500 ΔDL peaks showed the presence of the motif compared with 448/500 for KLF3. The DBD motif was slightly different to the KLF3 and ΔDL motifs and a more limited number of peaks (52/500) showed the presence of this motif. The reduction in the number of motifs in the ΔDL and DBD peaks may suggest that the specificity of these proteins has been compromised to some extent by the mutations introduced. In other words, the mutations appear to be reducing the discrimination in binding, but overall we see the retention of a consistent CACCC-like consensus sequence across the three samples (Supplementary Figure S13), which is presumably dependent on direct contacts between the ZF domain and DNA.

## DISCUSSION

Here, we have reported the genome-wide occupancy of KLF3 and defined the consensus sequence for *in vivo* bound KLF3 in MEFs. ChIP-seq data have previously been published for two other KLF family members; KLF1 in erythroid cells (6,39) and KLF4 in embryonic stem cells (7). KLFs are, therefore, one of the few families where the *in vivo* binding specificity of different family members can be compared (albeit in different cell types). There is a high degree of similarity between the emerging consensus DNA motifs of KLF1, KLF3 and KLF4 (Figure 4A–C) consistent with the high conservation in their ZF DBDs. Thus, the ZF DBD clearly plays a significant role in restricting the binding of KLF proteins to CACCC-like binding sites.

On the other hand, the biological roles of these three factors differ considerably. KLF1 drives erythroid maturation, KLF4 regulates pluripotency in stem cells and controls cell cycle progression in other contexts, whereas KLF3 functions in erythroid and B-cell development and adipogenesis (10,32,40,41). Just as the biological functions of these factors are diverse, so are their occupancy profiles (6,7,39). The differences between the genome-wide occupancy reported for KLF1, KLF3 and KLF4 may arise from multiple factors, including the fact that the proteins have been studied in different cell types, with different areas of open chromatin and possibly different cofactors, but also from the finding that regions outside their DBD can contribute to *in vivo* specificity.

We used a deletion and a point mutation in the N-terminus to test the contribution of non-DNA-contact regions to *in vivo* specificity and found that the mutations had a profound effect on binding *in vivo*. In general, deletion of the entire N-terminus, leaving only the ZF domain, significantly reduced binding, and mutation of the CtBP-contact motif by the two amino acid substitution reduced binding to a lesser extent. However, the actual profiles were complex, with examples of some regions where the mutants bound as well as wild-type and some where they bound better, as well as the more wide scale reduction in binding at many locations. The ΔDL and DBD mutants retained the preference for typical CACCC-like motifs, consistent with the fact that they were still relying on an intact ZF DBD. This observation is consistent with a recent genome-scale analysis of mammalian TF binding, which demonstrated that the DBD largely determines the DNA-binding consensus sequence (42). However, we also found that the stringency of binding and peak heights were often reduced compared with the full length protein, which may be in part a consequence of a reduced affinity of *in vivo* binding. Nevertheless, the results argue strongly that these N-terminal domains, hitherto thought to be dispensable for DNA-binding *in vitro*, are of considerable importance *in vivo*.

One hypothesis to explain this observation and the related finding that different KLF family members with different N-terminal domains bind different genes is that these KLFs and the mutants might differ in their ability to contact cofactor proteins that somehow enhance binding or increase the specificity of binding. KLF3 and KLF4, for example, bind CtBP but KLF1 does not (40,43). The KLF3 ΔDL construct that cannot bind CtBP only differs from wild-type KLF3 by the mutation of two amino acids making the observed changes in occupancy remarkable. KLF3 and ΔDL both bind to DNA with similar affinity *in vitro* [Supplementary Figure S2 and (11)], suggesting that binding differences *in vivo* may be attributable to contextual factors such as the presence of CtBP. KLF1 recruits entirely different cofactors including CBP/p300 (44), and the difference in these cofactors may explain different specificities.

**Figure 7.**
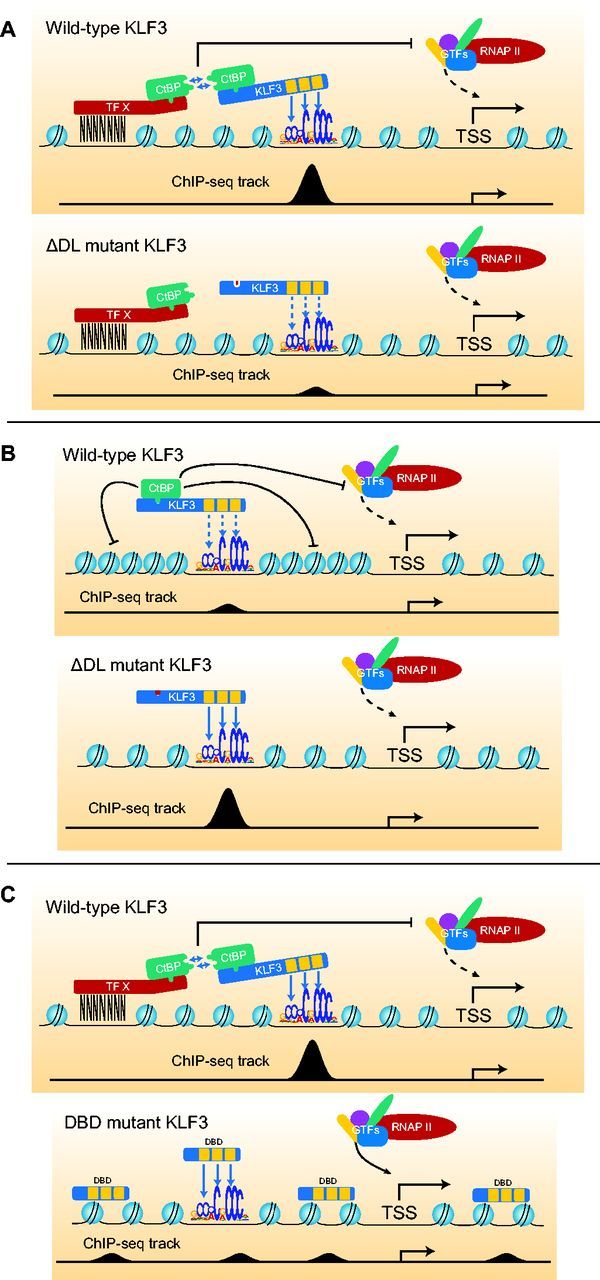
Simple models offer potential explanations for observed changes in occupancy. (**A**) CtBP is known to dimerize and can associate with >30 other mammalian TFs (45). It is possible that such interactions may stabilize wild-type KLF3 at certain genomic regions. (**B**) CtBP can modify chromatin domains via recruitment of a range of histone modifying enzymes. CtBP’s action at some regulatory elements may reduce occupancy by making chromatin less permissive. When KLF3 cannot properly recruit CtBP due to the ΔDL mutation, occupancy may increase as a result of chromatin being more open. (**C**) The DBD mutant shows higher DNA binding *in vitro* and also lacks the N-terminal domain that recruits CtBP. These two changes may lead to a decreased level of DNA-binding specificity with a concomitant increase in DNA-binding affinity potentially explaining the reduced occupancy observed genome-wide.

How CtBP contact may alter binding specificity *in vivo* is not currently clear but several direct and indirect effects may be at play. Most simply, one should note that CtBP is capable of self-associating and contacting >30 other vertebrate TFs (45). It may, therefore, act as a bridging molecule linking KLF3 to other DNA-bound TFs and enhancing targeting to specific loci already occupied by these factors (Figure 7A). In this way, the CtBP-binding motif may be important for directing KLF3 to specific sites and loss of the motif could result in loss of binding those sites. It may be particularly relevant that the CtBP-binding mutant appears to have particularly lost the ability to target promoter regions, regions where additional TFs may well be bound.

Indirect effects may account for the curious observation that the CtBP-contact point mutant actually bound better to certain loci. It is important to recall that KLF3 is a transcriptional repressor that appears to shut down chromatin domains by recruiting CtBP. However in *Klf3*^−^*^/^*^−^ cells rescued with the KLF3 CtBP-contact point mutant, these regions of chromatin would not be shut down and may remain open and accessible (Figure 7B). It is possible that the KLF3 mutant then has additional access to these loci, as they are more open rather than because the loss of CtBP contact facilitates KLF3’s ability to target specific loci. Figure 5C provides a good example of such a circumstance. Here, ΔDL has acquired a new binding specificity that occurs at a region where there is nucleosome aggregation in WT fibroblasts (DNase-seq track shows a low level of tags at this newly acquired peak) but which may be open in the ΔDL rescued cell line.

Finally, it is also likely that KLF3 genomic occupancy may be influenced by its participation in *cis*-regulatory modules, and to investigate this we examined KLF3 peaks for enrichment of other TFs binding motifs (Supplementary Figure S10). Our analysis of KLF3 promoter peaks revealed an association with binding sites for ETS family members (46) and CCAAT binding proteins (47), both of which are themselves known to be enriched at promoters. In addition, we also observed an enrichment of consensus sites for E2F proteins. Although a direct association between KLF3 and E2F has not been demonstrated, it is known that KLF1 regulates expression of both factors (6,48), suggesting potential for the regulation of shared target genes. At intronic and intergenic regions, we found similar binding sites in close proximity to KLF3 peaks, with notable enrichment of the AP1 consensus. Both KLF5 and KLF6 have previously been shown to interact with the AP1 protein c-Jun (49,50). Binding sites for RUNX proteins are also present in these regions and the interaction of RUNX and AP-1 is supportive of combinatorial binding to multicomponent *cis*-regulatory elements by these factors (51,52).

The occupancy of DBD is reduced many fold at most peaks, again particularly at gene promoters. It is difficult to interpret whether these data signify a loss of binding overall or a spreading of binding across more regions of the genome, giving lower peaks on average. We observe that DBD binds DNA more strongly *in vitro* (Supplementary Figure S2) and have also found that progressive deletion of the N-terminus of KLF3 increases in *vitro* DNA-binding affinity (Supplementary Figure S12). This increased affinity for DNA may lead to increased promiscuity by DBD and less specific binding *in vivo* (Figure 7C). If DBD’s binding affinity for DNA was increased, it may be redirected to what would usually be lower affinity sites, resulting in low occupancy of a greater number of genomic regions. This trend would result in a large reduction in peak height at KLF3 targets sites. Supporting this notion is the increased level of background in the RT-PCR negative controls in locations where KLF3 is not normally bound (Supplementary Figure S5A). Also of note is that the related protein KLF1 is known to have an autoinhibitory domain immediately N-terminal to the ZF region (53). This domain inhibits DNA-binding *in vitro* by interacting in *cis* with the DBD.

The observation that regions outside the ZF DBD of KLF3 are required for proper *in vivo* DNA binding is unexpected but fits with the converse observation that certain TFs retain functions even when their DBD is mutated. That is, these factors also appear to have regions outside their DBDs that contribute to localizing them to their target genes. For example, an SCL/TAL1 mutant with a non-functional DBD has been shown to partially rescue a knockout phenotype in hematopoietic cells (54). ChIP-seq revealed that the DBD mutant could still occupy ∼20% of the binding sites that were bound by the wild-type protein (3,55).

Similarly, studies on GATA-1 have recently revealed how cofactors can influence *in vivo* DNA-binding specificity. GATA-1 occupancy was shown to be dependent on its interaction with the cofactor friend of GATA-1 (FOG-1). A GATA-1 mutant carrying a non-functional binding domain for FOG-1 displayed a different occupancy profile than wild-type protein (2). In the absence of FOG-1, GATA-1 occupies mast cell specific genes and forced expression of FOG-1 can displace GATA-1 from these targets. Again, the precise mechanisms by which FOG-1 alters GATA-1 specificity are not yet clear.

Although we do not yet fully understand the mechanisms that determine the specificity of KLF3, it is clear that regions of the protein outside of the ZFs do influence targeting. In general, deletion of the entire N-terminus, significantly reduced binding and mutation of the CtBP-contact motif reduced binding to a lesser extent. However, the dependence on N-terminal domains for proper specificity is complex, with instances where the mutants showed similar binding to wild-type and other instances where binding was lost or gained. The finding that non-DBDs can affect KLF3 occupancy in such a manner has broader implications for understanding how TFs function *in vivo*. The ZF domain is the most common DBD in higher organisms, and a large number of proteins show a high level of conservation with KLFs, including SP factors, the GLI family, TFIIIA and others. Thus, it is likely that the specificity of other factors may also be dependent on non-DBDs. Finally, understanding how these additional domains operate may help advance the design of yet more effective and specific artificial DNA-binding proteins (56,57).

## ACCESSESSION NUMBERS

The ChIP-seq and microarray data from this article is available from the Gene Expression Omnibus (GEO) under accession numbers GSE44748 and GSE44744.

## SUPPLEMENTARY DATA

Supplementary Data are available at NAR Online.

## FUNDING

National Health and Medical Research Council and the Australian Research Council. Australian Postgraduate Award Scholarships (to J.B., C.A. and M.K.). Funding for open access charge: The University of New South Wales.

*Conflict of interest statement*. None declared.

## Supplementary Material

Supplementary Data
