# Supplementary material for: Regions outside the DNA-binding domain are critical for proper in vivo specificity of an archetypal zinc finger transcription factor
Source: Nucleic Acids Res. 2013 Oct 6;42(1):276–89. doi: 10.1093/nar/gkt895 (PMC3874204; doi:10.1093/nar/gkt895)
Supplement: Supplementary Data [file supp_42_1_276__index.html]

Regions outside the DNA-binding domain are critical for proper in vivo specificity of an archetypal zinc finger transcription factor — Regions outside the DNA-binding domain are critical for proper in vivo specificity of an archetypal zinc finger transcription factor — Supplementary Data 

# Regions outside the DNA-binding domain are critical for proper *in vivo* specificity of an archetypal zinc finger transcription factor

## Supplementary Data

files

**Files in this Data Supplement:**

- Supplementary Data - pdf file
- Supplementary Data - xlsx file
- Supplementary Data - xlsx file
