# Supplementary material for: Regions outside the DNA-binding domain are critical for proper in vivo specificity of an archetypal zinc finger transcription factor
Source: Nucleic Acids Res. 2013 Oct 6;42(1):276–89. doi: 10.1093/nar/gkt895 (PMC3874204; doi:10.1093/nar/gkt895)
Supplement: Supplementary Data [file supp_gkt895_nar-00984-f-2013-File009.pdf]

Supplementary Data

Regions outside the DNA-binding domain are critical for proper *in vivo* specificity of an archetypal zinc finger transcription factor.

Jon Burdach, Alister P. W. Funnell, Ka Sin Mak, Crisbel M. Artuz, Beeke Wienert, Wooi F. Lim, Lit Yeen Tan, Richard C. M. Pearson, Merlin Crossley.

Supplementary Figures

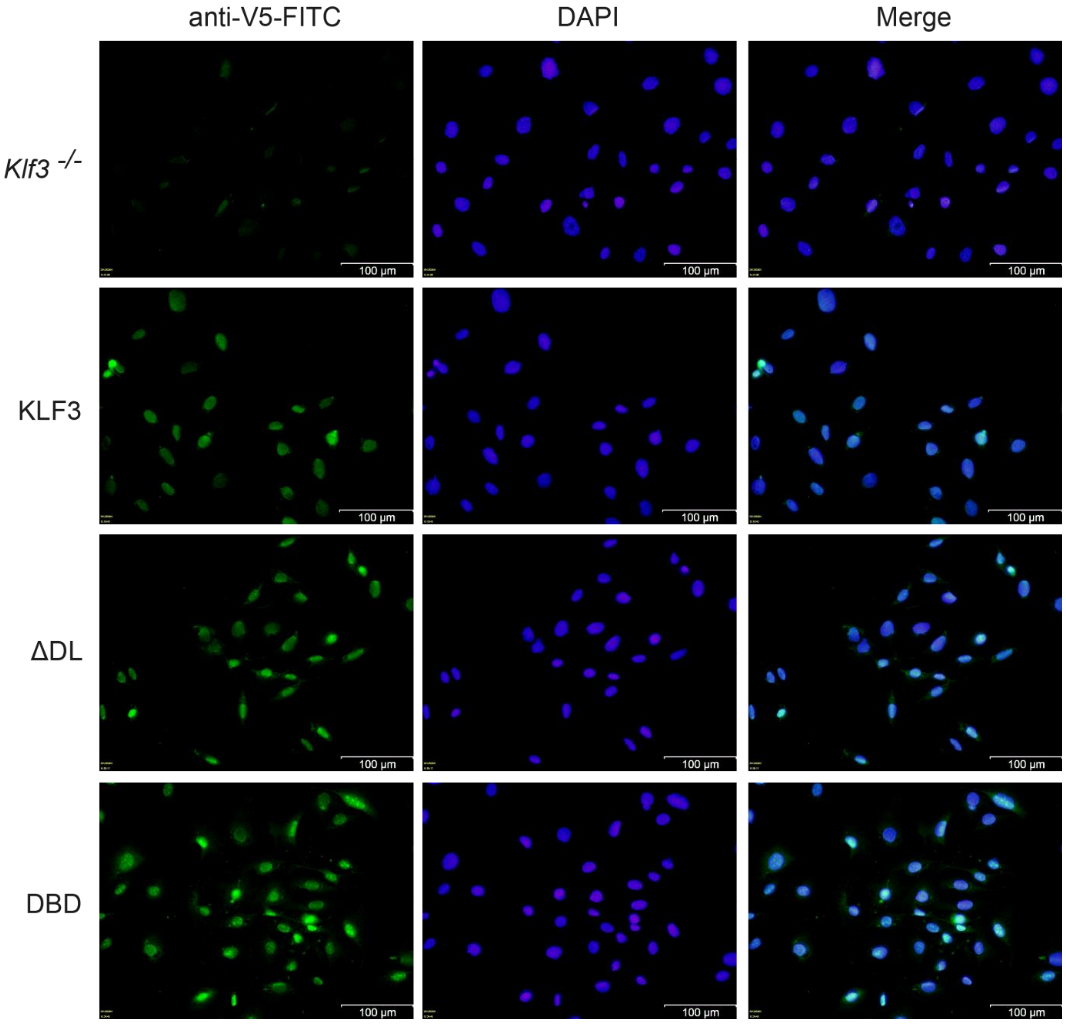

Supplementary Figure 1. Immunofluorescence confocal micrograph showing subcellular localisation of KLF3 and KLF3 mutant proteins. Anti-V5 FITC conjugated antibody is shown in green, while nuclear DAPI staining is blue.

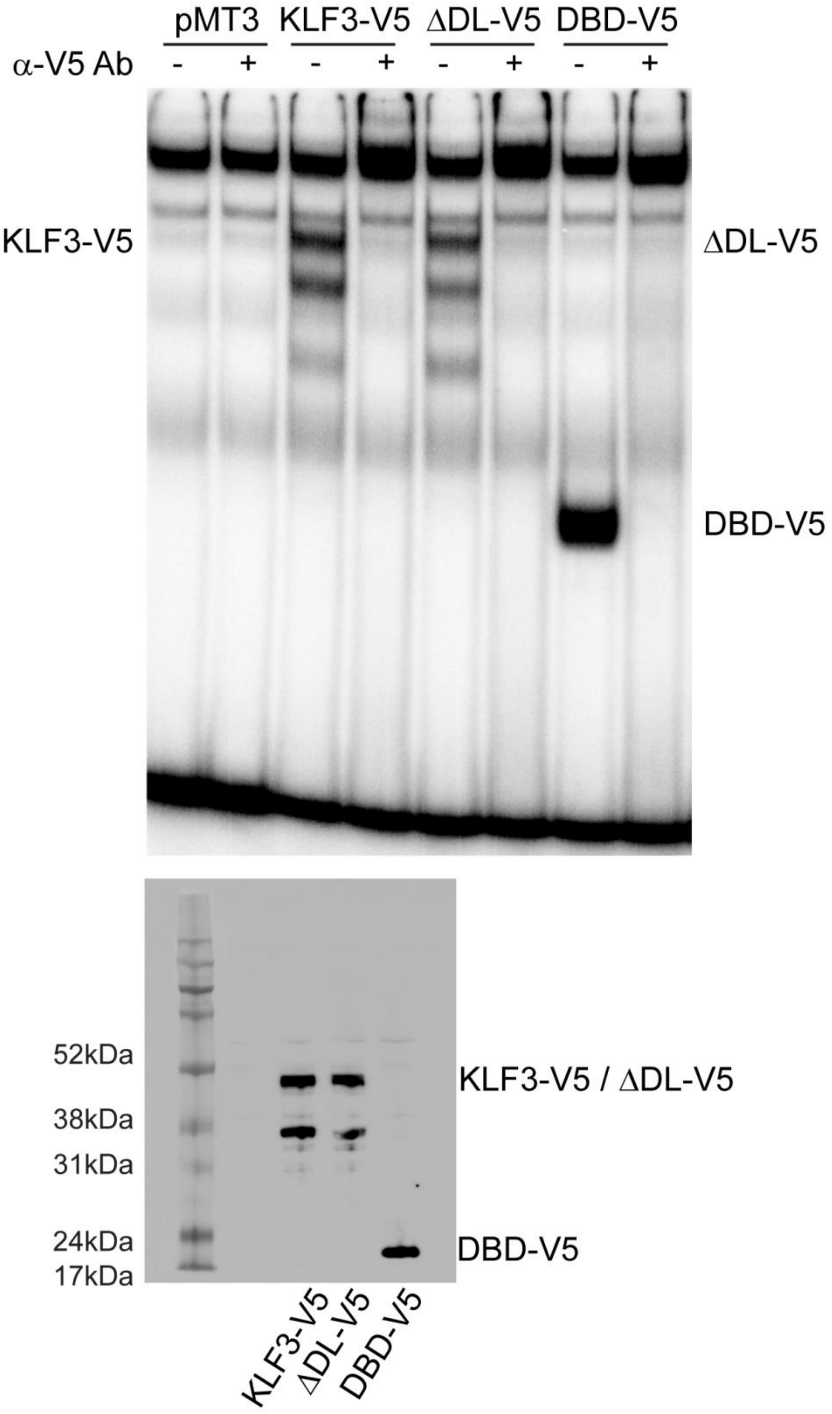

Supplementary Figure 2. EMSA showing binding of KLF3-V5, ΔDL-V5 and DBD-V5 to DNA. Constructs were expressed from pMT3 in 3T3-fibroblasts and binding of nuclear extracts to a *Klf8* CACCC-box probe assessed (1). KLF3-V5 and ΔDL-V5 show equivalent binding, while DBD-V5 binds more strongly (upper panel). Supershifts were performed with an anti-V5 antibody. Western blotting, also with an anti-V5 antibody, confirmed equal expression of all three constructs (lower panel). Probe sequences are given in Supplementary Table 2)

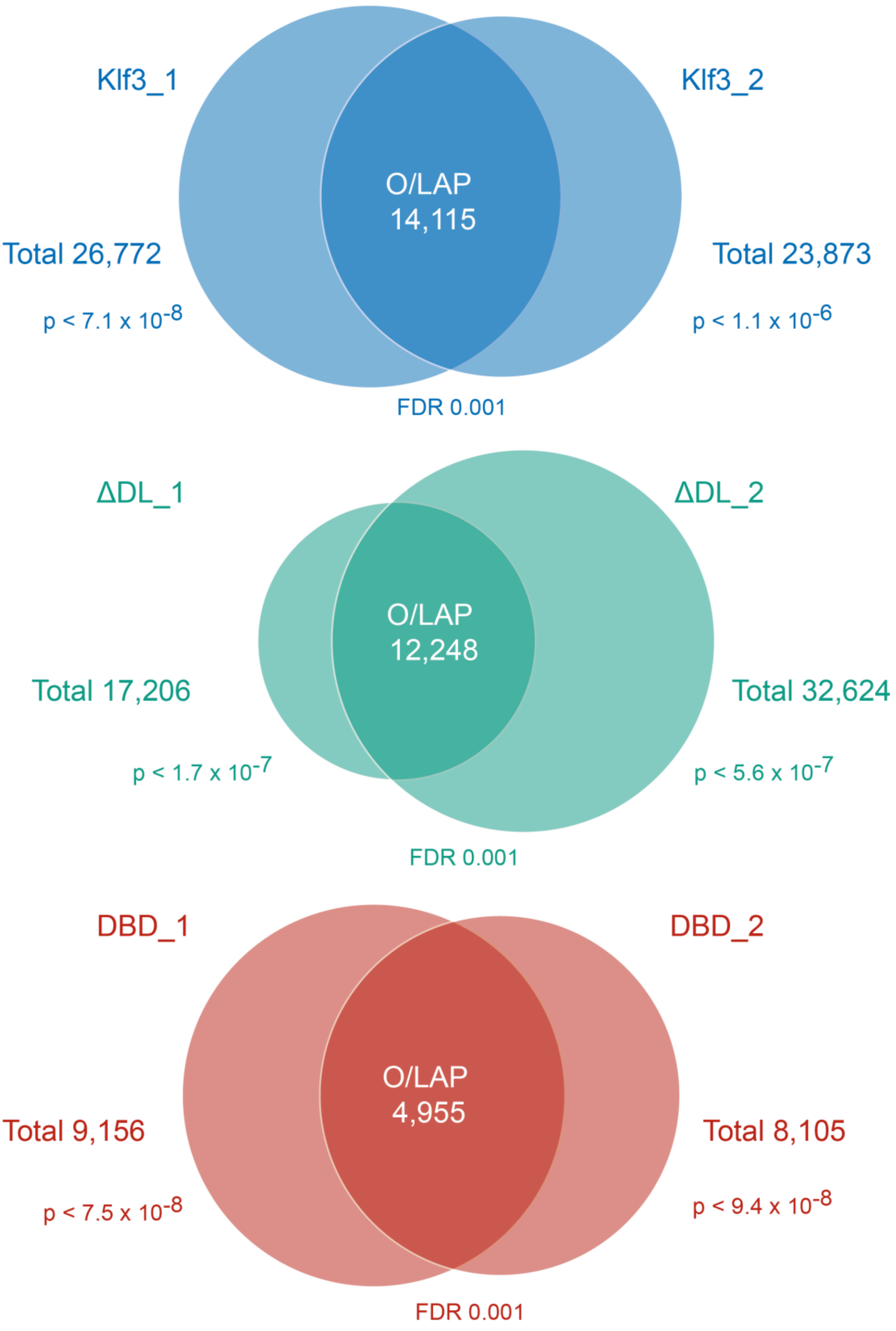

Supplementary Figure 3. Overlap of peaks called for each KLF3 ChIP-seq replicate. FDR cut-offs and p-values are given for each replicate. Venn diagrams are proportional.

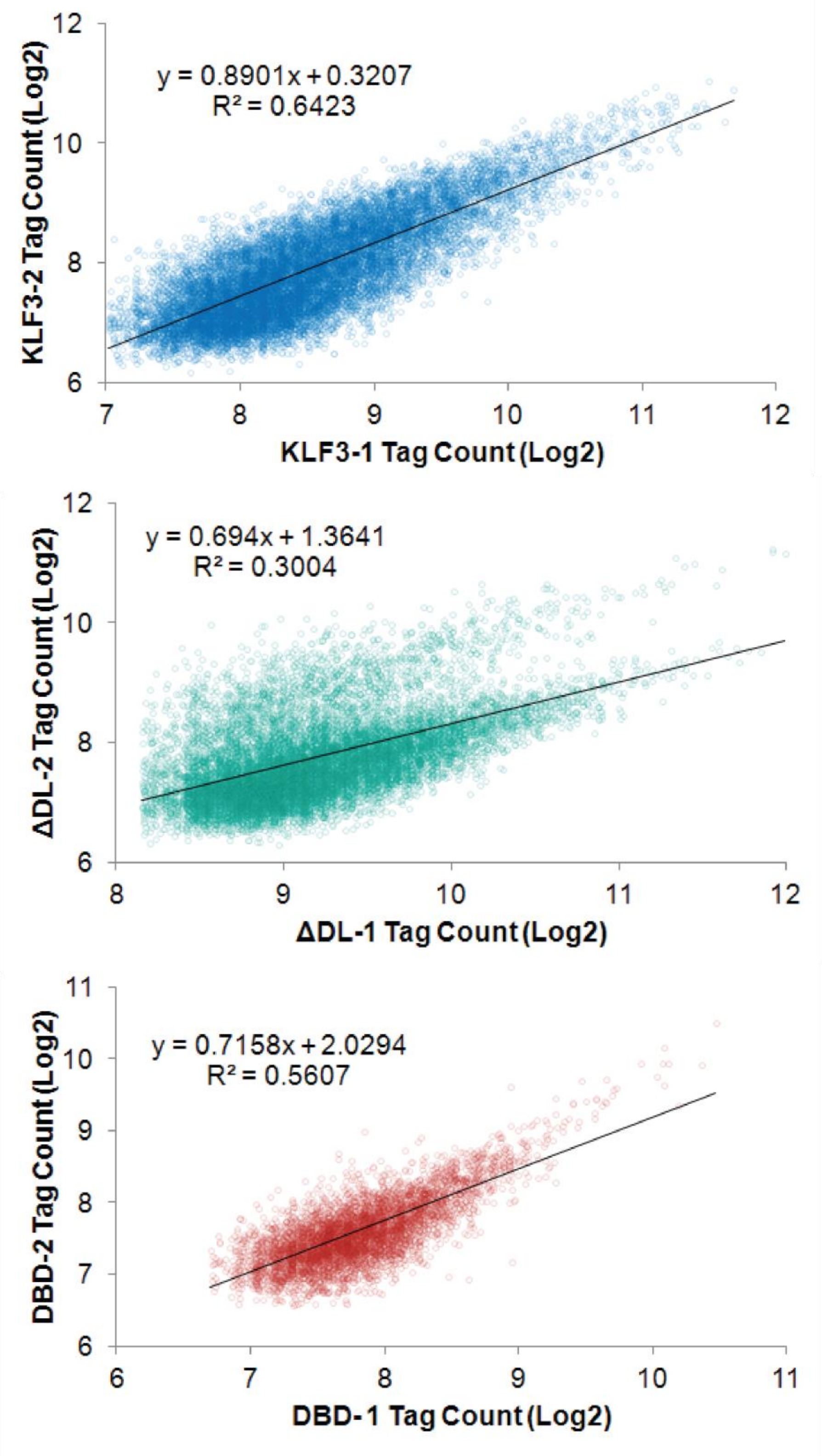

Supplementary Figure 4. Correlation between replicates at overlapping peaks. The overlapping peak regions between replicates were analysed to determine their correlation based on normalized tag counts within 400 bp of each peak centre.

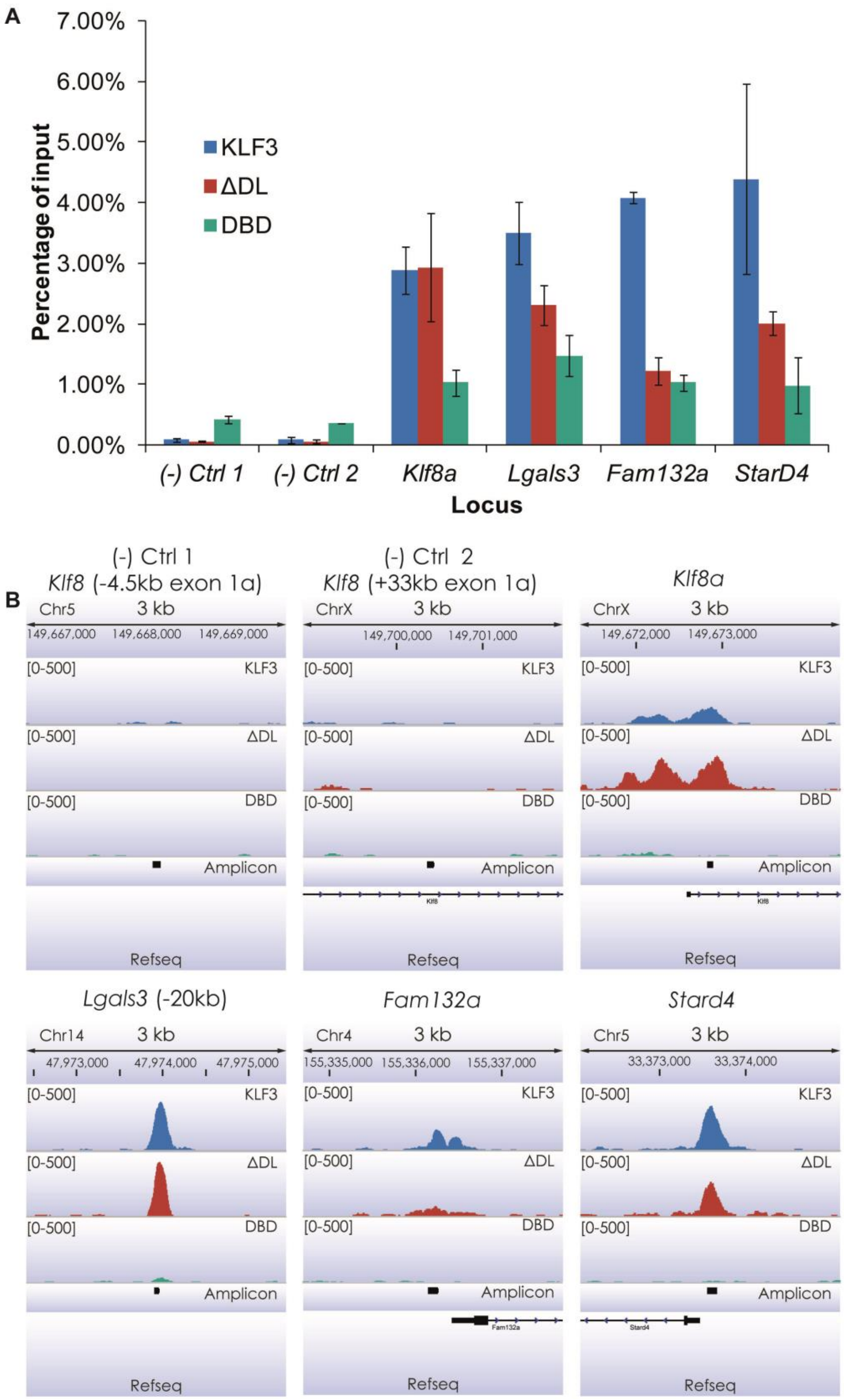

Supplementary Figure 5. Verifying ChIP-seq peaks by ChIP-PCR. (A) Selected peaks amplified by semi-quantative real-time PCR. (B) ChIP-seq tracks of the control peaks amplified in A. The location of the qPCR amplicons is indicated by the black boxes.

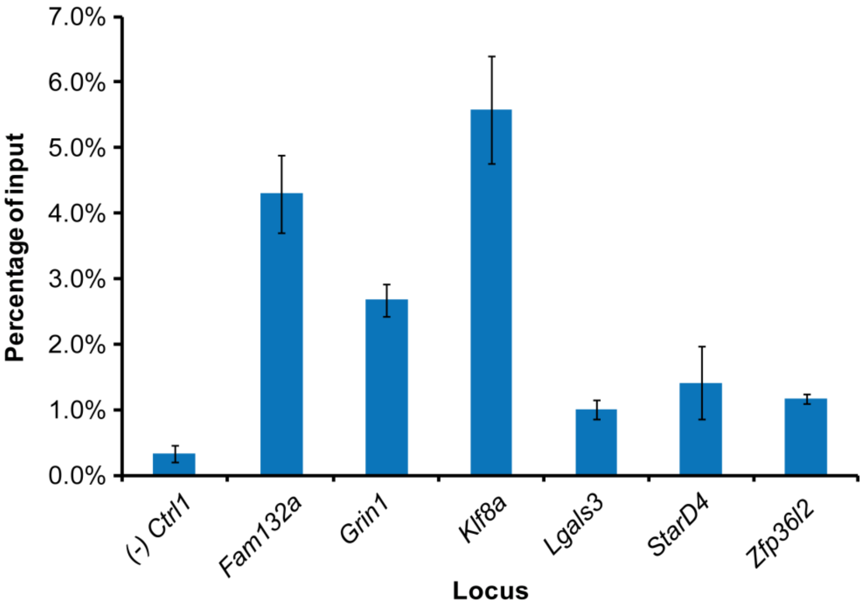

Supplementary Figure 6. Verifying that endogenous KLF3 and KLF3-V5 occupy the same genomic loci. Binding of endogenous KLF3 was examined in wildtype MEFs by ChIP. Shown are sites identified by ChIP-Seq as being bound by KLF3-V5. Data are the mean of three independent ChIP experiments; error bars represent standard error of the mean. Primer sequences for ChIP-PCR are given in Supplementary Table 3.

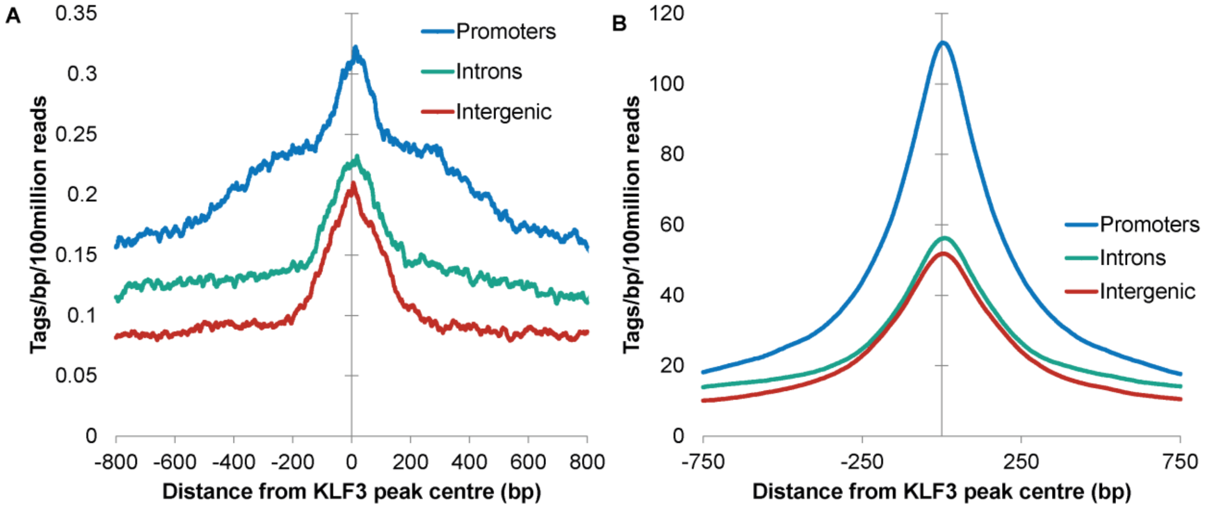

Supplementary Figure 7. Conservation and nucleosome depletion around KLF3 binding sites. (A) Cumulative sequence conservation at single nucleotide resolution across all KLF3 peaks at promoters, intronic and intergenic regions. (B) Cumulative distribution of nucleosome depletion as measured by DNase-seq at single nucleotide resolution across all KLF3 peaks at promoters, intronic and intergenic regions. The DNase-seq dataset was produced by the Stamatoyannopoulos laboratory at the University of Washington from mouse lung fibroblasts and was released under the ENCODE consortium (2,3).

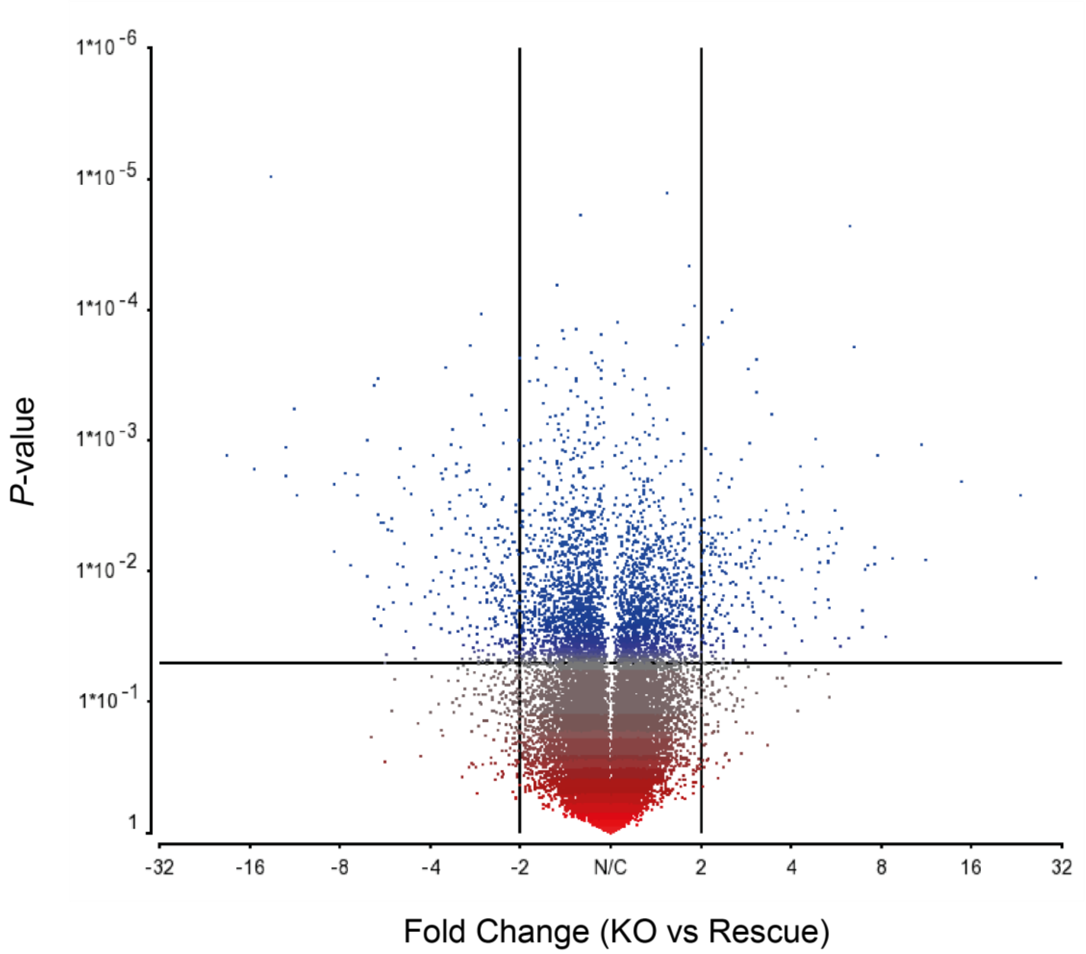

Supplementary Figure 8. Volcano plot of changes in gene expression on KLF3 rescue as measured by expression microarray. Affymetrix Mouse Gene ST microarrays were performed in duplicate on RNA extracted from *Klf3*$^{-/-}$ MEF cells and the same cell line rescued with *Klf3-V5*. Vertical lines indicate a greater than 2.0 fold difference between the two cell lines. The horizontal bar shows a *P*-value cut-off of 0.05.

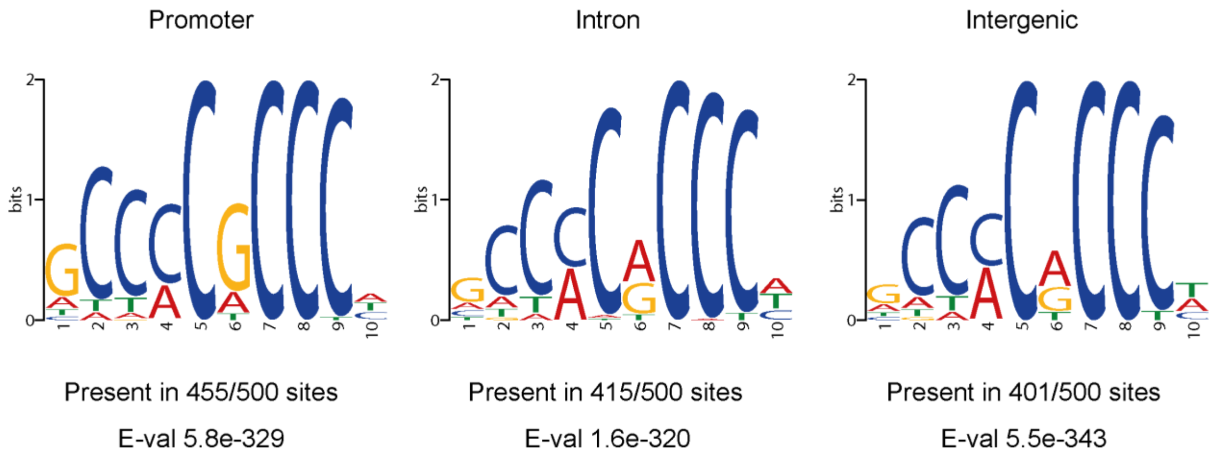

Supplementary Figure 9. *De novo* KLF3 motifs derived from KLF3 peaks in different genomic regions. Motif discovery was performed using MEME (4) on the 100 bp of the top 500 peaks in each region ranked by peak height. The frequencies of motifs are indicated below each position weight matrix.

A

Promoter				
Motif	Name	*P*-value	KLF3 peaks with motif	Background sequences with motif
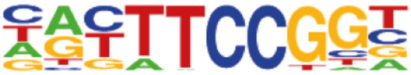	ETS family	1e-323	30.05%	9.18%
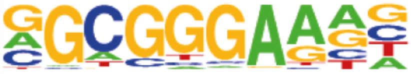	E2F family	1e-40	20.71%	13.24%
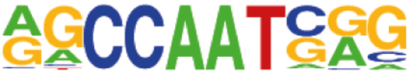	CCAAT binding proteins	1e-166	20.24%	7.16%
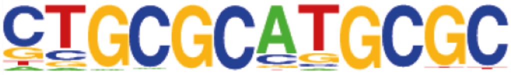	NRF1	1e-83	12.08%	4.64%

B

Intronic				
Motif	Name	*P*-value	KLF3 peaks with motif	Background sequences with motif
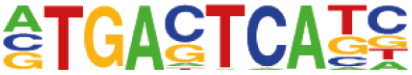	AP-1	1e-604	32.58%	6.14%
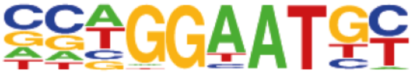	TEF family	1e-98	17.44%	7.62%
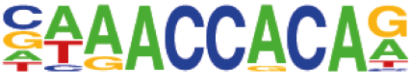	RUNX family	1e-76	15.09%	6.90%

C

Intergenic				
Motif	Name	*P*-value	KLF3 peaks with motif	Background sequences with motif
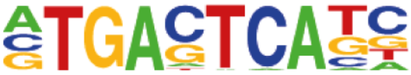	AP-1	1e-686	33.29%	6.57%
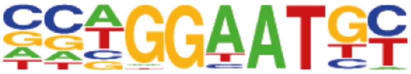	TEF family	1e-83	16.76%	8.26%
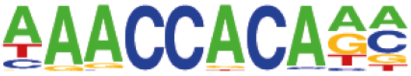	RUNX family	1e-65	18.82%	10.64%

Supplementary Figure 10. Co-occurrence of known transcription factor motifs enriched in KLF3 promoter (A), intronic (B), or intergenic peaks (C). The enrichment of known motifs at KLF3 peaks in various genomic regions was established using HOMER (5). Background sequences with equivalent di-nucleotide composition to the KLF3 peaks were also selected and the presence of the designated motifs in these background sequences is also given.

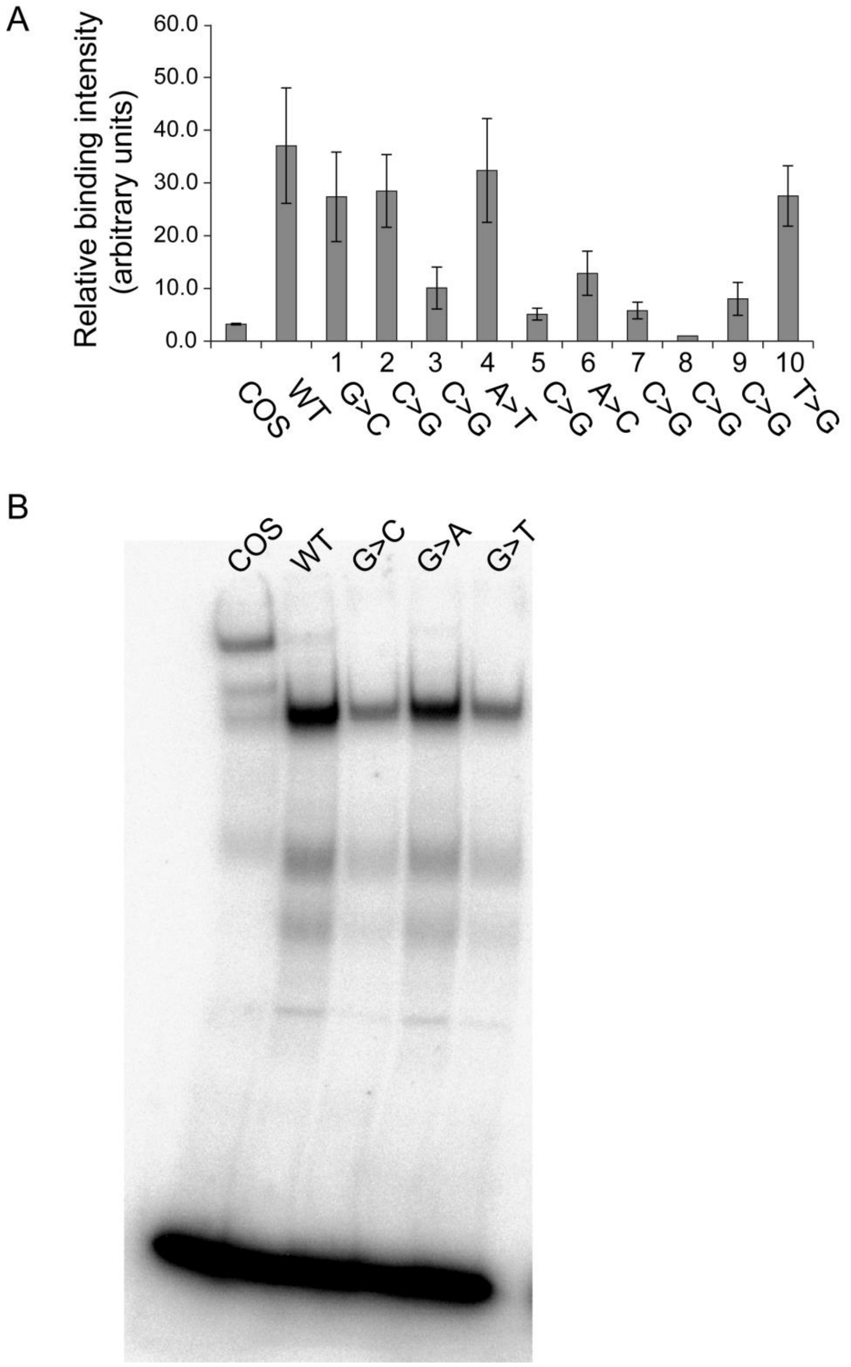

Supplementary Figure 11. (A) Relative contribution of each residue in the KLF3 consensus sequence to DNA-binding affinity. The relative binding intensity of KLF3 to probes containing point mutations in a sequence derived from the *β-globin* promoter (6) was examined by EMSA. The x-axis gives the position of each mutation and the base substitution. The numbering of the consensus maps to the binding motif in Figure 4A and a representative EMSA is shown in Figure 4F. Band intensities were determined using ImageJ v1.47 and are the average of two independent assays. Error bars show the standard error of the mean. **(B) Importance of position 1 of the KLF3 ChIP-Seq consensus for DNA-binding.** EMSA showing binding of KLF3 to a series of *β-globin* CACCC probes in which the G at position 1 was mutated to each of the three possible alternatives. COS cells were transfected with pMT3-KLF3. COS and WT lanes contain wildtype *β-globin* probe; COS lane contains nuclear extracts from cells transfected with empty pMT3 vector. Probe sequences are given in Supplementary Table 2.

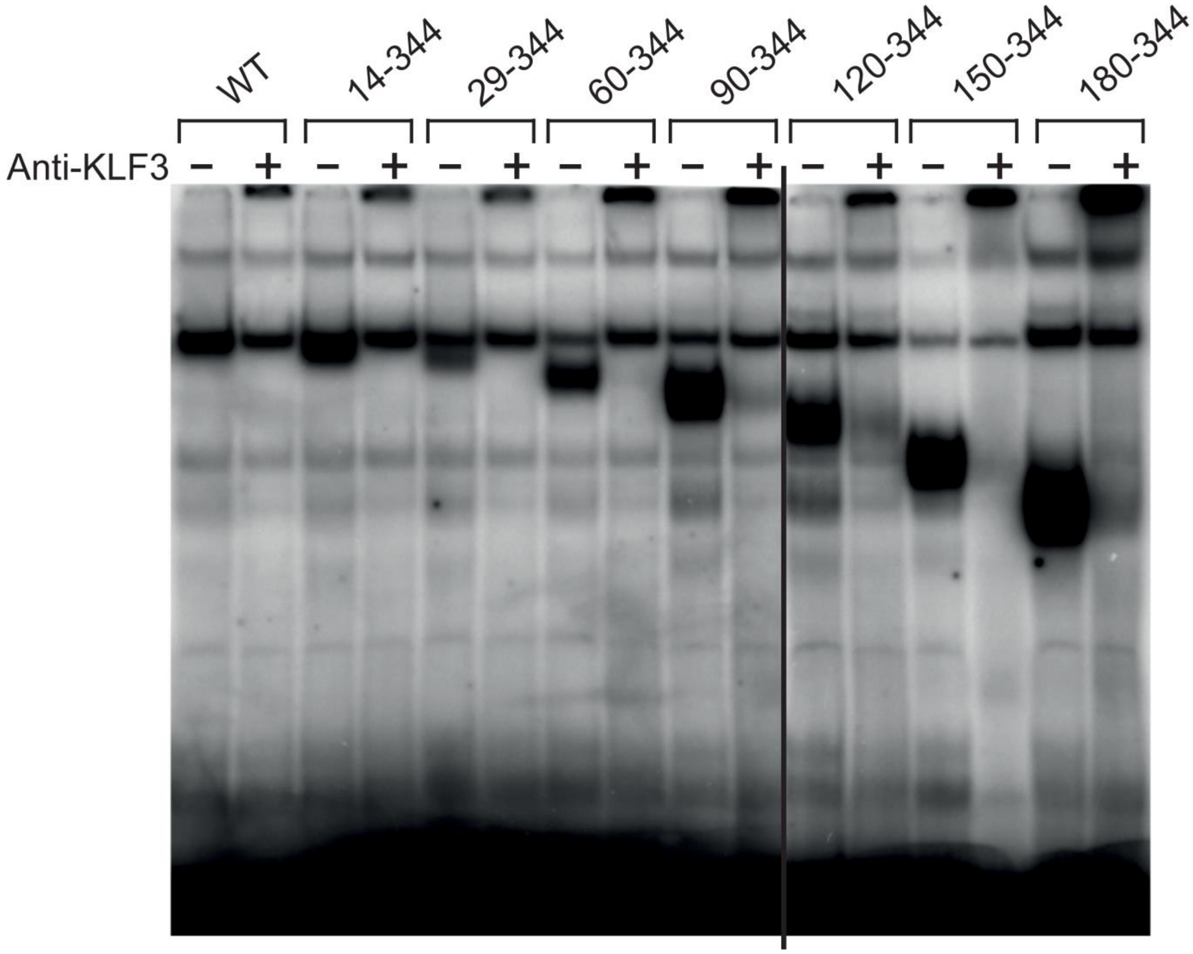

Supplementary Figure 12. Electrophoretic mobility shift assay (EMSA) on progressive N-terminal deletions of KLF3. Truncated mutants were expressed in COS cells and EMSAs were carried out as previously described using a probe from the *β-globin* locus (Supplementary Table 2) (6). A solid black line is included to indicate the splicing of two separate gels.

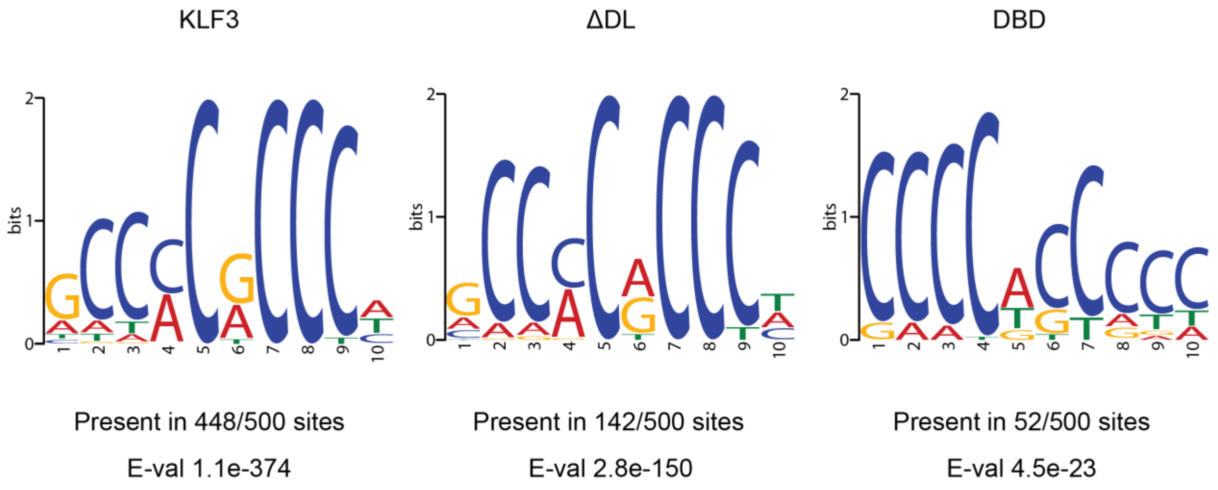

Supplementary Figure 13. Motif differences between KLF3, ΔDL and DBD peaks. *De novo* motif discovery was performed on the central 100 bp of the top 500 peaks ranked by peak height using MEME (4). Note that the frequency at which the motif occurs in the top 500 peaks is given below each motif.

Supplementary Tables

Note: Supplementary Tables 5 and 6 are available as excel spreadsheets and can be downloaded separately.

Supplementary Table 1. Oligonucleotide sequences for real time RT-PCR

Description	Primer sequence 5’ → 3’
18S rRNA-F	CAC GGC CGG TAC AGT GAA AC
18S rRNA-R	AGA GGA GCG AGC GAC CAA
Klf3-V5-F	CTGGGATCAAACCTTTCCAG
Klf3-V5-F	CCAGCAGAGGGTTAGGGATA

Supplementary Table 2. EMSA probe sequences.

Description	Oligo sequence 5’ → 3’
Klf8 sense	GGGCCCGCCCCACCCCCTCCT
Klf8 anti-sense	AGGAGGGGGTGGGGCGGGCCC
β-globin sense	TAGAGCCACACCCTGGTAAG
β-globin anti-sense	CTTACCAGGGTGTGGCTCTA
β-globin 1G>C sense	TAGACCCACACCCTGGTAAG
β-globin 1G>C anti-sense	CTTACCAGGGTGTGGGTCTA
β-globin 1G>T sense	TAGATCCACACCCTGGTAAG
β-globin 1G>T anti-sense	CTTACCAGGGTGTGGATCTA
β-globin 1G>A sense	TAGAACCACACCCTGGTAAG
β-globin 1G>A anti-sense	CTTACCAGGGTGTGGTTCTA
β-globin 2A>G sense	TAGAGGCACACCCTGGTAAG
β-globin 2A>G anti-sense	CTTACCAGGGTGTGCCTCTA
β-globin 3C>G sense	TAGAGCGACACCCTGGTAAG
β-globin 3C>G anti-sense	CTTACCAGGGTGTCGCTCTA
β-globin 4A>T sense	TAGAGCCTCACCCTGGTAAG
β-globin 4A>T anti-sense	CTTACCAGGGTGAGGCTCTA
β-globin 5C>G sense	TAGAGCCAGACCCTGGTAAG
β-globin 5C> G anti-sense	CTTACCAGGGTCTGGCTCTA
β-globin 6A>C sense	TAGAGCCACCCCCTGGTAAG
β-globin 6A>C anti-sense	CTTACCAGGGGGTGGCTCTA
β-globin 7C>G sense	TAGAGCCACAGCCTGGTAAG
β-globin 7C>G anti-sense	CTTACCAGGCTGTGGCTCTA
β-globin 8C>G sense	TAGAGCCACACGCTGGTAAG
β-globin 8C>G anti-sense	CTTACCAGCGTGTGGCTCTA
β-globin 9C>G sense	TAGAGCCACACCGTGGTAAG
β-globin 9C >G anti-sense	CTTACCACGGTGTGGCTCTA
β-globin 10T>G sense	TAGAGCCACACCCGGGTAAG
β-globin 10T>G anti-sense	CTTACCCGGGTGTGGCTCTA

Supplementary Table 3. Primer sequences for ChIP

Locus (F/R)	Primer sequence 5’ → 3’
Klf8 -4.5kb exon 1a-F (Ctrl 1)	GGTTTCTGAGACCTAACACTTCACACT
Klf8 -4.5kb exon 1a-R	CCATTTAGTCATCCAGCGAACAA
Klf8 +33kb exon 1a-F (Ctrl 2)	AACCTGGGTGCCTCCTTGTA
Klf8 +33kb exon 1a-R	TCATGCCTTTGACTTTAGTGCTTT
Klf8 +0.25kb exon 1a-F (Klf8a)	CCAGCTCGTGCACACTGAA
Klf8 +0.25kb exon 1a-R	GAAGCCTTAACATCAGGAGTGGAA
Lgals3-(-20kb)-F	TGGAAAAACACCCGTGCCTCTGA
Lgals3-(-20kb)-R	CAGTGCCTACGCCCAGATGACTC
Fam132a-prom-F	GATTCGCTTCCCTGGAGGTGTGG
Fam132a-prom-R	GCCCAGTCTCTGGTCTCCTCTCT
Stard4-prom-F	TCCAGCCACAGCCAATCA
Stard4-prom-R	TACTCCCGCTAACACCCCA
Grin1-F	GCCCCAAAAGTACAGATCTTCT
Grin1-R	TGTGGGCTGGAATTTGTGT
Zfp36l2-F	GCATTGGCGCGTTTCTC
Zfp36l2-R	GCGCTCAAGTCCTGTTTACTG

Supplementary Table 4. Summary of ChIP-seq reads mapped.

INPUT		IP	
Sample	Mapped Reads	Sample	Mapped Reads
Klf3_IN-1	70,287,321	Klf3_IP-1	40,296,064
Klf3_IN-2	90,772,480	Klf3_IP-2	47,015,286
ΔDL_IN-1	72,730,391	ΔDL _IP-1	20,189,146
ΔDL _IN-2	83,493,299	ΔDL _IP-2	35,576,092
DBD_IN-1	75,370,264	DBD _IP-1	42,535,264
DBD _IN-2	82,543,532	DBD _IP-2	46,292,414
Total	475,197,287	Total	231,904,266

Supplementary Table 5. Spreadsheet showing annotated KLF3, ΔDL and DBD peak locations and peak heights (see attached excel file).

Supplementary Table 6. Spreadsheet showing microarray data including intensities and significance testing by ANOVA. Spreadsheet showing genes dysregulated more than 2-fold on KLF3 rescue that also exhibit a promoter peak. (see attached excel file).

Supplementary References

1. Eaton, S.A., Funnell, A.P., Sue, N., Nicholas, H., Pearson, R.C. and Crossley, M. (2008) A network of Kruppel-like Factors (Klfs). Klf8 is repressed by Klf3 and activated by Klf1 in vivo. *J Biol Chem*, **283**, 26937-26947.
2. Encode Project Consortium. (2011) A user's guide to the encyclopedia of DNA elements (ENCODE). *PLoS biology*, **9**, e1001046.
3. Neph, S., Stergachis, A.B., Reynolds, A., Sandstrom, R., Borenstein, E. and Stamatoyannopoulos, J.A. (2012) Circuitry and dynamics of human transcription factor regulatory networks. *Cell*, **150**, 1274-1286.
4. Bailey, T.L. and Elkan, C. (1994) Fitting a mixture model by expectation maximization to discover motifs in biopolymers. *Proceedings / ... International Conference on Intelligent Systems for Molecular Biology ; ISMB. International Conference on Intelligent Systems for Molecular Biology*, **2**, 28-36.
5. Heinz, S., Benner, C., Spann, N., Bertolino, E., Lin, Y.C., Laslo, P., Cheng, J.X., Murre, C., Singh, H. and Glass, C.K. (2010) Simple combinations of lineage-determining transcription factors prime cis-regulatory elements required for macrophage and B cell identities. *Mol Cell*, **38**, 576-589.
6. Crossley, M., Whitelaw, E., Perkins, A., Williams, G., Fujiwara, Y. and Orkin, S.H. (1996) Isolation and characterization of the cDNA encoding BKLF/TEF-2, a major CACCC-box-binding protein in erythroid cells and selected other cells. *Mol Cell Biol*, **16**, 1695-1705.
